# Using focused ultrasound to modulate microglial structure and function

**DOI:** 10.3389/fncel.2023.1290628

**Published:** 2023-12-18

**Authors:** Sarina Grewal, Elisa Gonçalves de Andrade, Rikke Hahn Kofoed, Paul M. Matthews, Isabelle Aubert, Marie-Ève Tremblay, Sophie V. Morse

**Affiliations:** ^1^Department of Bioengineering, Imperial College London, London, United Kingdom; ^2^Department of Brain Sciences, Imperial College London, London, United Kingdom; ^3^Neuroscience Graduate Program, Division of Medical Sciences, University of Victoria, Victoria, BC, Canada; ^4^Division of Medical Sciences, University of Victoria, Victoria, BC, Canada; ^5^Department of Neurosurgery, Department of Clinical Medicine, Aarhus University, Aarhus, Denmark; ^6^Center for Experimental Neuroscience-CENSE, Department of Neurosurgery, Aarhus University Hospital, Aarhus, Denmark; ^7^Hurvitz Brain Sciences Research Program, Biological Sciences, Sunnybrook Research Institute, Toronto, ON, Canada; ^8^UK Dementia Research Institute, Imperial College London, London, United Kingdom; ^9^Department of Laboratory Medicine and Pathobiology, Temerty Faculty of Medicine, University of Toronto, Toronto, ON, Canada; ^10^Axe Neurosciences, Centre de recherche du CHU de Québec-Université Laval, Québec, QC, Canada; ^11^Department of Molecular Medicine, Université Laval, Québec, QC, Canada; ^12^Department of Biochemistry and Molecular Biology, University of British Columbia, Vancouver, BC, Canada

**Keywords:** focused ultrasound, blood-brain barrier, modulation, microglia, glia, functional effects, neurodegeneration

## Abstract

Transcranial focused ultrasound (FUS) has the unique ability to target regions of the brain with high spatial precision, in a minimally invasive manner. Neuromodulation studies have shown that FUS can excite or inhibit neuronal activity, demonstrating its tremendous potential to improve the outcome of neurological diseases. Recent evidence has also shed light on the emerging promise that FUS has, with and without the use of intravenously injected microbubbles, in modulating the blood-brain barrier and the immune cells of the brain. As the resident immune cells of the central nervous system, microglia are at the forefront of the brain’s maintenance and immune defense. Notably, microglia are highly dynamic and continuously survey the brain parenchyma by extending and retracting their processes. This surveillance activity aids microglia in performing key physiological functions required for brain activity and plasticity. In response to stressors, microglia rapidly alter their cellular and molecular profile to help facilitate a return to homeostasis. While the underlying mechanisms by which both FUS and FUS + microbubbles modify microglial structure and function remain largely unknown, several studies in adult mice have reported changes in the expression of the microglia/macrophage marker ionized calcium binding adaptor molecule 1, and in their phagocytosis, notably of protein aggregates, such as amyloid beta. In this review, we discuss the demonstrated and putative biological effects of FUS and FUS + microbubbles in modulating microglial activities, with an emphasis on the key cellular and molecular changes observed *in vitro* and *in vivo* across models of brain health and disease. Understanding how this innovative technology can modulate microglia paves the way for future therapeutic strategies aimed to promote beneficial physiological microglial roles, and prevent or treat maladaptive responses.

## 1 Drug delivery to the brain: a longstanding challenge

### 1.1 The blood-brain barrier

**FIGURE 1:**
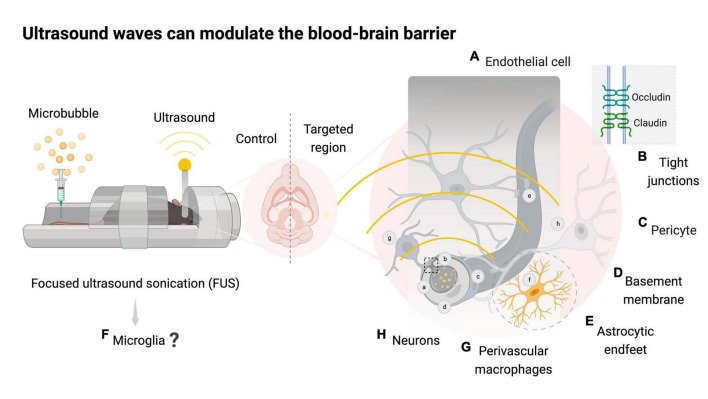
Focused ultrasound sonication with or without microbubbles can modulate the neurovascular unit. Schematic representation of mice exposed to magnetic resonance imaging-guided focused ultrasound with microbubbles (FUS + microbubbles) targeting one brain hemisphere and using the contralateral side as a control. Bubble oscillations caused by ultrasound waves can modulate the neurovascular unit (NVU). The NVU is comprised of blood-brain barrier (BBB) components such as endothelial cells **(A)**, junctional proteins **(B)**, pericytes **(C)**, basement membrane **(D)**, astrocytic endfeet **(E)**, microglia **(F)**, and perivascular macrophages **(G)** that collectively support neuronal function **(H)**. FUS + microbubbles leads to a transient downregulation of tight junction proteins such as occludin and claudin, increasing the paracellular transport rates of the BBB. The mechanical stress and the evasion of blood-derived molecules into the brain following FUS + microbubbles are thought to trigger an acute inflammatory process in the targeted area. Created with BioRender.com.

The brain is confined to a dynamic and tightly controlled environment responsible for homeostasis and health, known as the blood-brain barrier (BBB) (Figure 1). The BBB is a semi-permeable and multicellular barrier that provides an optimal environment for neuronal and synaptic activity through the regulation of ion channels such as Na^+^, K^+^, Ca^2 +^, and Cl^–^ (Wu et al., 2023). Together with a network of transporters, receptors and cells, the BBB allows the transfer of essential molecules such as oxygen and glucose from the blood to the brain. The BBB also serves protective functions by preventing the passage of pathogens, toxins and peripheral immune cells to the central nervous system (CNS). While the restrictive properties of the BBB are essential to brain health and function, they also limit the entry of many promising therapeutics that could be used to treat CNS disorders, such as brain tumors, ischemic stroke, epilepsy, and neurodegenerative diseases (Pardridge, 2005).

### 1.2 The neurovascular unit

The BBB is maintained by the neurovascular unit, which is comprised of neurons, endothelial cells, basement membrane, pericytes, astrocytes, perivascular macrophages and microglia (Segarra et al., 2019; Figure 1). The endothelial cells (Figure 1A) of cerebral capillaries are tightly sealed by junctional proteins (Figure 1B), which restrict the extravasation of blood-borne molecules into the brain (Engelhardt, 2003). Pericytes (Figure 1C) surround the endothelial cells and provide structural support by releasing signaling molecules such as vascular endothelial growth factors and transforming growth factor-β, which contribute to the stability of tight junctions (Armulik et al., 2010). The basement membrane (Figure 1D) is a type of extracellular matrix composed of glycoproteins, including laminins and collagen type IV, which regulate the movement of cells and molecules, and promote the structural stability of the epithelium and endothelial tissue (Leclech et al., 2020). Astrocytes (Figure 1E) are the most abundant glial cells in the CNS and provide structural support to the *glia limitans*, a second protective barrier that allows astrocytes to tightly cover pericytes and endothelial cells with their endfeet (Abbott et al., 2006). Apart from their structural role, astrocytes play an essential role in protecting neuronal function by monitoring the activity of synapses and regulating osmotic balance in the CNS (Abbott et al., 2006). Microglia (Figure 1F), the resident immune cells of the CNS, constitute the remaining portion of the *glia limitans* and play a pivotal role in forming and supporting the neurovascular unit (Šimončičová et al., 2022).

### 1.3 Microglia

Microglia are highly dynamic cells (Nimmerjahn et al., 2005; Kettenmann et al., 2011) that regulate the function of other immune, glial and neuronal cells in the CNS (Šimončičová et al., 2022). For instance, microglia promote synaptic remodeling through partial (i.e., trogocytosis) or full (i.e., phagocytosis) elimination of synaptic elements (Weinhard et al., 2018). In addition, microglia can physically separate synaptic elements (i.e., stripping) interrupting synaptic transmission, can remodel the extracellular matrix to stimulate post-synaptic development, and can participate in the formation of dendritic spines by releasing neurotrophic factors (Nguyen et al., 2020). Microglia have a diverse range of cellular states that vary according to the context, for example, the biological sex, life stage and brain region (Paolicelli et al., 2022). As a result of this diversity, in this review we outline the contextual information available (e.g., sex, brain region, stage of life examined) when reporting microglial results, a policy highly encouraged by experts (Paolicelli et al., 2022). When exposed to brain insults, microglia can proliferate, migrate, change their transcriptome, proteome, metabolome, morphology, and ultrastructure to participate in the immune response of the brain, notably through phagocytosis of debris, infected or apoptotic cells, and release of soluble factors such as cytokines, chemokine and neurotrophic factors (Tremblay et al., 2011; Bellenguez et al., 2022; Šimončičová et al., 2022).

Beyond these functions, microglia significantly interact with cells of the neurovascular unit. It is estimated that ∼30% of microglial cell bodies are associated with capillaries across multiple brain regions in mice such as the cerebral cortex, thalamus, and hippocampus (Bisht et al., 2021). In the cerebral cortex of adult male mice as well as in aged and middle-aged humans (female and male), the processes of microglia are repetitively in contact with most of the vasculature for periods lasting between 5 and 15 min (Bisht et al., 2021; Császár et al., 2022). Császár et al. (2022) showed that microglial processes, which express the purinergic receptor P2Y G-protein coupled 12 (P2Y12R), interact with most astrocytic endfeet in the mouse cerebral cortex. Besides endfeet, P2Y12R-positive processes also interact with pericytes, in addition to 15% of endothelial cell surfaces in cortical blood vessels of adult female and male mice (Császár et al., 2022). The impact of microglial-BBB contacts in adulthood is an emerging field, with evidence that it aids in the regulation of blood vessel dilation and constriction, as well as BBB repair (Haruwaka et al., 2019; Bisht et al., 2021; Császár et al., 2022). In addition to their homeostatic role, microglia are involved in the pathophysiology of several neurodegenerative disorders such as Alzheimer’s disease (AD), Parkinson’s disease and amyotrophic lateral sclerosis, for instance by sustaining inflammation (Bachiller et al., 2018). Therefore, targeting microglia is a promising therapeutic strategy to prevent the onset and progression of these diseases.

## 2 Focused ultrasound: a window into the brain

### 2.1 Therapeutic challenges due to the blood-brain barrier

The BBB hinders access to intravenous treatments that modulate microglia (Šimončičová et al., 2022). This barrier has previously been overcome in the clinic through invasive intracranial injections requiring brain surgery or intravenous injections of therapeutics and hyperosmotic solutions at high dosages, which can lead to side-effects (Gerstenblith et al., 2012; Mendell et al., 2017; Castle et al., 2020). Importantly, Hynynen et al. (2001) demonstrated that the permeability of the BBB can be increased temporarily by using focused ultrasound (FUS) with intravenously injected microbubbles. FUS is an incisionless technology that uses highly localized ultrasound waves penetrating deep into tissue at high spatial resolution (Fini and Tyler, 2017; Figure 1). With a minimally invasive and highly precise BBB targeting, FUS provides advantages over other CNS-related technologies, such as transcranial magnetic stimulation and direct current stimulation, as well as current neurological therapies such as deep-brain stimulation, radiation, and surgery (Fini and Tyler, 2017). The early application of FUS dates to the 1940s, when it was first demonstrated to induce thermal ablation in the liver tissue of mice (Lynn et al., 1942). Moreover, Fry et al. (1958) first documented the neuromodulation potential of FUS, in which neural activity in the thalamus of cats was reversibly inhibited. Subsequently, researchers observed changes in the permeability of the BBB within and in proximity to the regions targeted with FUS, which established the foundation for current and ongoing research into the therapeutic potential of this strategy (Bakay et al., 1956; Ballantine et al., 1960).

### 2.2 Microbubbles influence the outcomes of FUS

The pressure oscillation of FUS causes the expansion and contraction or collapse of intravenously administered microbubbles, which results in a temporary increase in BBB permeability (Wonhye and Seung-Schik, 2021; Figure 1). Variations in the formulation of these microbubbles can influence the outcome of FUS treatments. In experiments conducted on adult male rats, factors such as shell type (e.g., lipid or protein) and differences in dosage (e.g., 300 or 450 μl/kg) did not seem to affect the cavitation levels consistently (Yang et al., 2009; Silburt et al., 2017; Wu et al., 2017). However, the diameters of microbubbles, such as 1–2 μm, 4–5 μm and 6–8 μm, resulted in different outcomes on BBB modulation at various acoustic pressures. At 1.5 MHz and 0.30 MPa, the 1–2 μm microbubbles failed to induce detectable BBB permeability (Samiotaki et al., 2012). In contrast, FUS with 4–5 and 6–8 μm microbubbles led to BBB modulation for an extended duration, over 24 h and 3 days, respectively (Samiotaki et al., 2012). Similar outcomes were observed in experiments comparing microbubbles of 1–2 and 4–5 μm diameter (Choi et al., 2009), as well as acoustic pressures of 0.3 MPa, 0.45 MPa and 0.6 MPa at 1.5 MHz (Wang et al., 2015). Notably, in clinical studies, the microbubble usage is adjusted to promote safe BBB modulation (Lipsman et al., 2018; Abrahao et al., 2019; Mainprize et al., 2019; Meng et al., 2021; Park et al., 2021).

### 2.3 Therapy delivery with FUS + microbubbles

While most clinical trials so far have targeted brain volumes without any intravenously injected therapies, FUS + microbubbles has been shown to promote the delivery of therapeutic agents in individuals with brain tumors, amyotrophic lateral sclerosis and Parkinson’s disease (Mainprize et al., 2019; Meng et al., 2021). As opposed to intracranial injections, where each injection site comes with tissue damage along the needle track, FUS can be applied to any brain region in rodents and humans without affecting adjacent areas, and this has the potential to confer a targeted yet widespread delivery of therapeutics (Felix et al., 2021; Park et al., 2021; Kofoed et al., 2022). Preclinically, FUS + microbubbles has demonstrated the ability to increase the permeability of several barriers between the blood and the CNS and has enabled the delivery of therapeutics to the brain, spinal cord, retina, and brain tumors (Weber-Adrian et al., 2015; Chang et al., 2017; Touahri et al., 2020). The range of therapeutics that have been delivered to the rodent brain with this technology is broad and includes small molecule drugs, recombinant proteins, nanoparticles, viral vectors, and stem cells, for the treatment of a large spectrum of animal models of neurological disorders such as AD, Parkinson’s disease, Huntington’s disease, spinal cord injury, and glioblastoma (Jordão et al., 2010; Burgess et al., 2014; Chang et al., 2017; Mead et al., 2017; Song et al., 2017; Xhima and Aubert, 2021; Kofoed et al., 2022).

### 2.4 FUS alone has important biological outcomes

**FIGURE 2:**
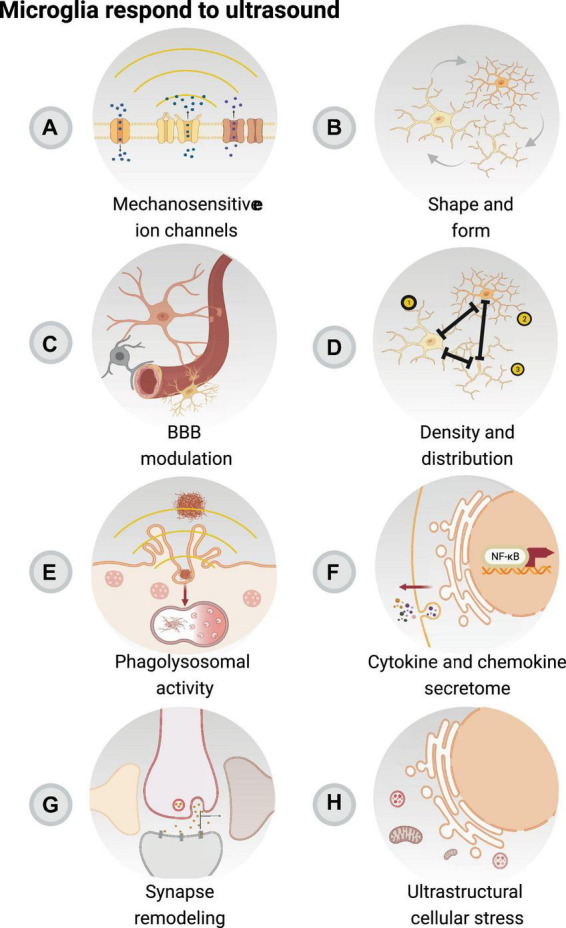
Focused ultrasound sonication with or without microbubbles can modulate microglial function. Microglia, as the resident immune cells of the brain, are involved in the acute inflammatory response resulting from focused ultrasound with or without microbubbles, in concert with remaining elements of the neurovascular unit. Focused ultrasound stimulation (FUS) alone is associated with reduced pro-inflammatory secretory activity of microglia and activation of mechanosensitive ion channels **(A)**. FUS with and without microbubbles are connected to changes in microglial morphology **(B)**, which may impact their interactions with the blood-brain barrier (BBB) **(C)** to regulate blood flow. In addition, changes in cell number, distribution **(D)**, phagolysosomal activity **(E)**, cytokine and chemokine secretion **(F)** are an outcome of FUS + microbubbles and FUS, notably modulating the surveilling area of microglia, crucial in their roles in regulating the immune response as well as glial and neuronal function **(G)**. Moreover, it is possible that the modulatory effect of FUS + microbubbles and FUS impacts the ultrastructure of microglia **(H)**, with relevant outcomes on markers associated with cellular stress. Created with BioRender.com.

The effects of FUS on the brain can be modulated by several parameters. Ultrasound waves are generated by a piezoelectric transducer that converts electrical signals into mechanical vibrations (Krishna et al., 2018). The input voltage and power to the transducer’s piezoelectric material determine the pressure of the emitted ultrasound waves, which controls the intensity (acoustic pressure per given volume) of the ultrasound treatment (Wonhye and Seung-Schik, 2021). The acoustic pressure of ultrasound waves can activate mechanosensitive ion channels and induce ruffling of the plasma and internal membranes, i.e., motile cell surface protrusions, thus impacting cell function (Yoo et al., 2018; Figure 2A). FUS, without intravenously injected microbubbles, has been shown to have neuromodulatory effects, activating *in vitro* rodent primary hippocampal and cortical Ca^2 +^ and Na^2 +^ mechanosensitive channels to cause influx of extracellular Ca^2 +^. Notably, high-intensity FUS has been shown to elicit action potentials in skin peripheral nerves via the stimulation of PIEZO2 in female mice (Hoffman et al., 2022). Moreover, FUS directed to the somatosensory cortex of healthy human adult volunteers, predominantly males, resulted in transient tactile sensations and sonication-specific evoked potentials (Lee et al., 2015). Clinical trials have also reported the efficacy of FUS as a neuromodulation tool for the treatment of epilepsy and neuropathic pain in human adult females and males with temporal lobe epilepsy or chronic and therapy resistant neuropathic pain (Stern et al., 2021; Gallay et al., 2023). Within the brain, microglia express multiple mechanosensitive ion channels and are likely to be affected by FUS acoustic radiation forces (Blackmore et al., 2023). For instance, activation of the mechanosensitive Ca^2 +^ channel PIEZO1 in microglia increased the number of ionized calcium-binding adapter molecule 1 (Iba1) positive (+) cells and their co-localization with amyloid beta (Aβ) plaques in the hippocampus of adult male 5xFAD mice, a model of AD pathology (Jäntti et al., 2022). Thus far, however, most microglial findings in the FUS field concentrate on the outcomes derived from BBB modulation in the presence of intravenous microbubbles.

## 3 Effect of FUS and FUS + microbubbles: the immune hypothesis

### 3.1 Evidence of infiltration of blood-derived molecules

From drugs of approximately 500 Da to antibodies of 150 kDa, or gene vectors of 4 MDa, FUS + microbubbles-induced BBB modulation provides a powerful tool to increase the entry of molecules into the brain, as demonstrated in rodents (Meng et al., 2021), non-human primates (Blesa et al., 2023) and humans undergoing clinical trials (Meng et al., 2021). Depending on the ultrasound parameters, the BBB permeability is increased to allow the passage of molecules of varying sizes into the brain (Chen et al., 2014). This increased permeability tendentially persists for several hours following FUS + microbubbles, although blood-derived components can linger in the brain parenchyma for several days (Todd et al., 2019). Multiple studies have reported that albumin (67 kDa), the most abundant protein found in the bloodstream, can enter the brain after FUS + microbubbles treatments when using specific ultrasound parameters (Alonso et al., 2010; Leinenga and Götz, 2015; Kovacs et al., 2017). For instance, in male rats targeting the cerebral cortex with FUS + microbubbles at a high pressure of 1.25 MPa and 1 MHz, increased levels of albumin extravasation 30 min after the treatment have been observed, which persisted up to 24 h (Alonso et al., 2010). In addition to albumin, immunoglobulins (Ig) are also abundant blood proteins. Increased extravasation of endogenous Ig occurs in animals treated with FUS + microbubbles, including wild-type mice and mouse models of AD pathology (TgCRND8, APP/PS1 and B6C3-Tg) (Kinoshita and Hynynen, 2005; Kinoshita et al., 2006; Raymond et al., 2008; Jordão et al., 2010, 2013; Dubey et al., 2020; Bathini et al., 2022). In both female and male mice overexpressing the human amyloid precursor protein (TgCRND8), an increase in endogenous IgG (150 kDa) and IgM (900 kDa) extravasation into the brain parenchyma has been observed between 4 h and 4 days after the cerebral cortex was targeted with 0.5 MHz FUS + microbubbles at 0.3 MPa (Jordão et al., 2013). Functionally, both albumin and immunoglobulins could act as chaperones to increase microglial phagocytosis (Bard et al., 2000; Brazil et al., 2000; Jordão et al., 2013; Leinenga and Götz, 2015; Sun et al., 2021), although further *in vivo* evidence would strengthen this statement. Overall, it is evident that blood-borne substances extravasating into the brain following FUS + microbubble treatments can promote specific microglial responses. Indeed, the stereotaxic injection of blood plasma into the brain of wild-type mice has been shown to elicit changes in the expression levels of genes associated with the detection of the blood clotting factors, such as fibrinogen, and phagocytosis upregulators, such as complement proteins in microglia (Mendiola et al., 2023). Here, increased levels of fibrinogen and complement induced the transcription of oxidative stress factors and a broad class of cytokines, such as type I interferon (Mendiola et al., 2023). A similar scenario could take place following FUS + microbubble treatments.

### 3.2 Evidence of cytokine production

Considering microglia as proficient immune surveillants, their interactions with blood-derived molecules and the mechanical stress exerted by FUS with and without microbubbles can result in an increased production of pro-inflammatory cytokines, influencing microglial activities. Cytokines are a heterogeneous group of signaling proteins secreted by cells of the CNS, including neurons, glia, and immune cells. During physiological events, cytokines regulate communication between cells, synaptic pruning and the release of neurotrophic and inflammatory factors that can have pro or anti-inflammatory effects, among other outcomes (Turner et al., 2014; Werneburg et al., 2017). In the context of injury or disease, cytokines can initiate and regulate the immune response by recruiting various CNS cells, including microglia, to promote tissue regeneration and the return to homeostasis (Turner et al., 2014). However, prolonged exposure to high levels of these proteins can result in neuronal damage and disease progression (Deng et al., 2010; Turner et al., 2014). Male rats with common peroneal nerve injury, a model of neuropathic pain, treated with FUS at an acoustic intensity of 8 W, were found to have a decreased protein expression of interleukin (IL)-6, IL-1β and tumor necrosis factor (TNF)-α, pro-inflammatory cytokines produced by immune cells which are involved in acute-phase inflammatory responses against infections and tissue damage (Hellman et al., 2021). Of note, IL-6, IL-1β and TNF-α can be released by microglia, and their elevated levels are associated with the onset of neuropathic pain (Zhao et al., 2017). FUS treatment alone can also modulate microglial cytokine production in mice and alleviate allodynia, a type of neuropathic pain, thereby providing a promising intervention to mitigate some of the symptoms associated with inflammation (Hellman et al., 2021). Similar outcomes were also reported in the substantia nigra of male rats challenged with 6-hydroxydopamine (6-OHDA), which models Parkinson’s disease pathology (Sung et al., 2022). In this context, 1 MHz FUS alone at 528 mW/cm^2^ reduced the protein levels of IL-1β and enhanced glial cell line-derived neurotrophic factor, a protein known for promoting the survival of dopaminergic neurons in Parkinson’s disease (Sung et al., 2022). This dual effect induced by FUS possibly plays a significant role in modulating microglial responses and their interactions with neurons, which are involved in the pathology of Parkinson’s disease. Furthermore, studies have reported that the reduction in the pro-inflammatory response with FUS is linked to an inhibition of the nuclear factor-kappa B (NF-κB) pathway (Chen et al., 2019; Yi et al., 2022). Intriguingly, BBB modulation studies using microbubbles with FUS have reported an upregulation of pro-inflammatory cytokines elicited by NF-κB activation (McMahon and Hynynen, 2017; McMahon et al., 2020; Ji et al., 2021). FUS + microbubbles emitted at 580 kHz were also shown to lead to increased gene expression of TNF-α and IL-1β in the treated brain quadrant of male rats (McMahon et al., 2020). Similar changes were reported by Ji et al. (2021) at 6 h after FUS + microbubbles, which increased IL-6 and IL-1β gene expression in male mice. These changes in the expression levels of cytokines could be associated with the stress exerted on the vascular endothelium by microbubble oscillations (McMahon and Hynynen, 2017). Notably, the outcomes of fluctuations in the levels of IL-6, IL-1β and TNF-α on microglia following FUS or FUS + microbubbles have not been clarified yet.

### 3.3 Evidence of chemokine production

Chemokines are chemoattractant cytokines, small signaling proteins that coordinate immune responses by regulating immune cell communication and directing their migration to targeted regions (Hughes and Nibbs, 2018). The infiltration of molecules and endothelial stress exerted by FUS + microbubbles can trigger the production of chemokines by various cells including microglia. In a study comparing different acoustic pressures at 1.1 MHz, FUS + microbubbles was found at 0.42 MPa, in contrast to 0.25 MPa, to drastically upregulate the gene expression of chemokine (C-C motif) ligand 2 (CCL2), ligand 12 (CCL12), and ligand 3 (CCL3), which are involved in directing microglia and macrophages to migrate toward affected brain regions (Ransohoff, 2009; Choi et al., 2022). Of note, the lower acoustic pressure of 0.25 MPa was sufficient to increase the permeability of the BBB without inducing damage or a high expression of chemokines (Choi et al., 2022). Additionally, a study documented that a single treatment of 589.636 kHz of FUS + microbubbles at 0.3 MPa in the cerebral cortex of adult female rats increased protein expression of damage-associated molecular patterns (DAMPs), resulting in an upregulation of chemokine (C-X-C motif) ligand 1 (CXCL1) and ligand 3 (CXCL3), which are associated with tissue repair and the recruitment of neutrophils, a type of white blood cell involved in the early stages of immune responses (Kovacs et al., 2017). DAMPs are signals released from damaged cells upon tissue injury and here it is likely that the high acoustic pressure and microbubble dose contributed to the increased expression of chemokines that led to the immune cell stimulation (Kovacs et al., 2017; Roh and Sohn, 2018). Following 1 MHz FUS + microbubbles at 0.5 MPa in mice with a melanoma brain tumor, increased gene expression levels of several chemokines have been observed. These chemokines coordinate innate immune cell migration, including CC12, CCL12, CXCL1 and an endothelial protein, intercellular adhesion molecule 1, which promotes the permeability of the BBB, allowing the migration of monocytes and lymphocytes (Curley et al., 2020). Ongoing efforts in the research community aim to establish FUS + microbubbles parameters that can safely modulate the BBB while minimizing brain tissue damage (O’Reilly and Hynynen, 2012; Xhima and Aubert, 2021; Xhima et al., 2022). However, it is important to note that mild and temporary inflammation can be beneficial to promote the clearance of debris which is required for tissue regeneration (Nathan and Ding, 2010). In this context, elevated levels of cytokines and chemokines following FUS + microbubbles may enhance the protective mechanisms of the immune system by attracting and modulating the functions of microglia, infiltrating macrophages and lymphocytes to the targeted area, ultimately promoting a better outcome for several neurological conditions.

### 3.4 Evidence of immune infiltration

#### 3.4.1 Macrophages

Along with inflammatory molecules, FUS + microbubbles can facilitate the infiltration of immune cells, such as neutrophils, macrophages and T lymphocytes, into specific brain regions, which can be beneficial to target brain tumors and other neurological diseases (Poon et al., 2021). The brain hosts two distinct populations of macrophages: microglia and CNS-associated macrophages (Sevenich, 2018; Dermitzakis et al., 2023). CNS-associated macrophages are predominantly present within the meninges, choroid plexus and perivascular space, where they protect the border regions of the CNS (Goldmann et al., 2016; Van Hove et al., 2019). In adult male rats treated with 1.5 MHz FUS + microbubbles at 2.45 MPa, intracerebral hemorrhage was observed 4 h post-treatment accompanied by an infiltration of macrophages, identified by integrin alpha M (CD11b) immunohistochemistry and MRI superparamagnetic iron oxide nanoparticles labeling (Liu et al., 2010). However, with treatment at 1.1 MPa no evident inflammatory response or brain hemorrhage was detected, suggesting that high acoustic pressure and tissue damage during FUS + microbubbles can induce macrophage infiltration (Liu et al., 2010). While macrophages can exhibit phenotypic characteristics differing from microglia, most FUS + microbubble studies have used markers that cannot distinguish between the two cell types, including Iba1, CD11b and the phagolysosomal marker cluster of differentiation (CD) 68 (Liu et al., 2010; Kovacs et al., 2017; Li and Barres, 2018; Pouliopoulos et al., 2021). Discrimination between macrophages and microglia can, however, be performed using multiomics approaches such as single-cell RNA sequencing, proteomics and epigenetics. For instance, *in vitro*, Zhang et al. (2019) reported an increased expression of anti-inflammatory-related genes such as arginase 1 (Arg1), peroxisome proliferator activated receptor gamma (PPAR-γ) and IL-4 involved in tissue regeneration, following 1.5 MHz ultrasound treatment of bone marrow-derived macrophages and raw264.7 cells, a murine derived macrophage-like cell line. Similarly, following ultrasound stimulation, human monocytic THP-1 macrophage-like cells treated with lipopolysaccharide (LPS) had decreased gene expression of pro-inflammatory of TNF-α and IL-8, and increased gene expression of IL-10 which promotes the resolution of inflammation, indicating that ultrasound can modulate cytokine expression in macrophages *in vitro* (Fontana et al., 2023). LPS-stimulated macrophage-like cells, obtained from U937 monocyte-like cells, treated with 38 kHz ultrasound at 250 mW/cm^2^ have been shown to significantly reduce the expression of pro-inflammatory cytokines *in vitro* (Iacoponi et al., 2023). In addition, primary mouse microglial cells cocultured in the presence of bone marrow-derived macrophages derived from male C57BL/6 mice significantly downregulated genes associated with inflammation, such as IL-6, IL-1β and TNF-α, thereby suggesting that in the context of FUS + microbubbles, the infiltration of macrophages and the cell-to-cell communication with microglia could result in a controlled and anti-inflammatory immune response (Greenhalgh et al., 2018).

#### 3.4.2 T cells

The adaptive immune system is made up of specialized T lymphocytes that can generate an effective immune response, notably in neurological diseases (Schetters et al., 2018). Like macrophages, T lymphocytes are rare in the healthy brain parenchyma and are primarily present in the meninges and choroid plexus (Hickey, 2001). The migration of T lymphocytes across the BBB is a tightly controlled process which is facilitated by the release of chemokines by both microglia and astrocytes, and through antigen presentation (Aloisi et al., 2000; Schetters et al., 2018). *In vitro*, primary microglial cells, isolated from SJL/J mice stimulated with Theiler’s murine encephalomyelitis virus, a model of viral infection, resulted in an increase in major histocompatibility complex class II and costimulatory molecules B7-1, associated with antigen presentation, thus leading to T lymphocyte activation (Olson et al., 2001). Following multiple FUS + microbubble treatments in a mouse model of glioma, the tumor microenvironment presented a significant increase in CD80+ antigen presenting cells, likely microglia, as well as lymphocytes, monocytes, and neutrophils compared to naïve animals (Zhang et al., 2023). Cancer cells can inhibit the functions of T lymphocytes, such as that of CD8 and CD4, which play a crucial role in recognizing and eliminating cancer cells, thereby creating a challenge in anti-tumor immune responses (Schreiber et al., 2011). FUS + microbubbles have been shown to enhance the delivery of immunotherapeutic agents to the brain, which has led to a favorable outcome on T lymphocyte activity and tumor growth (Chen et al., 2015; Ye et al., 2021; Sheybani et al., 2022). A single session of 0.5 MHz FUS + microbubbles at 0.7 MPa enhanced the delivery of IL-12 in a male C6 glioma rat model, which has previously been shown to elevate antitumor properties against glioma (Liu et al., 2002; Chen et al., 2015). Following the delivery of IL-12 with FUS + microbubbles, flow cytometry analysis reported an upregulation of T lymphocyte population, including CD3+ and CD4+, which correlated with impairment of tumor growth compared to IL-12 administration alone (Chen et al., 2015). Additionally, in a GL261 mouse glioma model, 1.5 MHz FUS + microbubbles at 0.43 MPa enhanced the delivery and colocalization of anti-programmed cell death-ligand 1 antibody with glioma cells which promoted antitumor effects by enabling T lymphocytes to target the cancer cells (Ye et al., 2021). Of note, the recruitment of T lymphocytes upon FUS + microbubbles is not only limited to gliomas. Liu et al. (2012) demonstrated that 0.5 MHz FUS + microbubbles pulsed at 1.4 MPa in subcutaneous CT-26 tumors in mice significantly reduced tumor growth by 34% and increased the infiltration of cytotoxic CD8+ T cells. In a breast cancer model, 1.15 MHz FUS thermal ablation at 6 MPa resulted in the migration of CD8+ cells within the tumor site, significantly slowing the tumor growth compared to the control (Cohen et al., 2020). Similar observations were also reported in melanoma and hepatocellular carcinoma cancer models (Bandyopadhyay et al., 2016; Ran et al., 2016). These results hold important implications for brain tumors as currently available treatments, such as chemotherapy and radiation, can cause functional impairment in immune cells (Merrick et al., 2005; Kaur and Asea, 2012). Therefore, treatment with FUS could enhance the immune response and offer long-term control of the disease. Notably, the role of microglia was not defined in the above-mentioned studies, however, it is known that microglia interact with T lymphocytes through antigen presentation (Schetters et al., 2018).

## 4 Microglia respond to FUS and FUS + microbubbles

### 4.1 Changes in morphology

Microglia constantly adapt their morphology to probe and sense their environment (Savage et al., 2019; Bosch and Kierdorf, 2022). On average, microglia extend and retract their processes at a rate of 1.5 μm per minute in mice (Nimmerjahn et al., 2005; Fuhrmann et al., 2010) and can increase their cell body size to enhance translation and traduction, as well as phagocytic turnover (Gonçalves de Andrade et al., 2022). The morphological states of microglia exist in a spectrum and are broadly classified as: ramified (normal soma, highly branched processes), ameboid (enlarged cell soma with filopodia), hypertrophic (large soma, short and thick processes), rod-shaped (small nuclei and bipolar processes), and dystrophic (fragmented) (Savage et al., 2019; Šimončičová et al., 2022). However, it is important to note that morphological analyses, albeit informative, are not standardized across research teams (Green et al., 2022) and represent one level of complexity of microglial function that should be complemented with other analyses (Paolicelli et al., 2022). Notably, both FUS and FUS + microbubbles are tied to morphological changes in microglia (Bobola et al., 2020; Silburt and Aubert, 2022; Table 1), providing insights into, for example, their surveillance activity and contribution to the *glia limitans* (Savage et al., 2019; Figure 2B). Acute FUS treatment for an hour in male 5XFAD mice resulted in an increased prevalence of hippocampal Iba1+ cells presenting ameboid or hypertrophic morphologies, e.g., a smaller aspect ratio (ratio of cell body over width) and higher roundness. Correspondingly, these cells were found to co-localize with Aβ plaques, in comparison with sham and non-treated hemispheres (Bobola et al., 2020). Considering that the mechano-sensitive PIEZO1 calcium channel in microglia detects Aβ stiffness, leading to cell clustering and Aβ compaction (Hu et al., 2023), the morphological responses after FUS could be linked to the mechanisms occurring after PIEZO1 activation through acoustic radiation.

**TABLE 1:** Effects of FUS and FUS + microbubbles on microglia.

Model	Brain region	Microbubbles (Y/N)	Parameters^a^	Changes in Microglia^b^	Measurement^c^	References
Alzheimer’s pathology (5xFAD mice)	Hippocampus	N	fc = 2 MHz; I = 190 w/cm^2^; PL = 400 us; PRF = 40 Hz; T = 5 s on/off for 1 h	↑ Iba1+ co-localization with Aβ	IF	Bobola et al., 2020
Alzheimer’s pathology (TgCRND8)	Right cortex	Y	fc = 0.5 MHz; P = 0.3 MPa; PL = 10 ms; PRF = 1 Hz; T = 120 s	↑ Iba1+ co-localization with Aβ clearance	IF	Jordão et al., 2013
Alzheimer’s pathology (rTG4510)	Hippocampus	Y	fc = 1.5 MHz; P = 0.45 MPa; PL = 6.7 ms; PRF = 10 Hz; T = 60 s	↑ Iba1+ co-localization with tau	IF	Karakatsani et al., 2019
Alzheimer’s pathology (C57BL/6)	Hippocampus	Y	fc = 835 kHz; PL = 10 ms; PRF = 2 Hz; T = 100 s	↑ Iba1+ co-localization with Aβ	IF	Sun et al., 2021
Alzheimer’s pathology (TgCRND8)	Hippocampus	Y	fc = 1.68 MHz; PL = 10 ms; PRF = 1 Hz; T = 120 s	↑ Iba1+ co-localization with Aβ	IF	Silburt and Aubert, 2022
Parkinson’s pathology (6-OHDA)	Right hemisphere	N	fc = 1 MHz; I = 528 mW/cm^2^; PL = 50 ms; PRF = 1 Hz; T = 15 min	↓ IL-1β	ELISA	Song et al., 2022
Multiple sclerosis (EAE)	Right hemisphere	Y	fc = 690 kHz; P = 260–270 kPa; PL = 10 ms; PRF = 2 Hz; T = 95 s	↑ Iba1+ TMEM119+	IHC	Schregel et al., 2021
Middle cerebral artery occlusion (ICR)	Not specific hemisphere	N	fc = 0.5 MHz; I = 120 mW/cm^2^; PL = 0.5 ms; PRF = 1,000 Hz; T = 300 ms	↑ Iba1+ arginase+ ↑ IL-10	IF RTqPCR	Wang et al., 2021
Inflammation (LPS)	Hippocampus and cortex	N	fc = 1 MHz; I = 528 mW/cm^2^; PL = 50 ms; PRF = 1 Hz; T = 15 m	↓ TNF-α, IL-1β ↑ BDNF	WB	Chen et al., 2019
Healthy	Left frontal cortex and right hippocampus	Y	fc = 548 kHz; P = 0.144 MPa (increases of 0.0008 MPa); PL = 10 ms; PRF = 0.5–0.6 Hz; T = 120 s	↑ Iba1+	IF	Sinharay et al., 2019
Healthy	Thalamus	Y	fc = 1.1 MHz; P = 0.25, 0.42 MPa; PL = 10 ms; PRF = 1 Hz; T = 120 s	↑ Iba1+ ↑ NK-κB	IF Transcriptomics	Choi et al., 2022
Healthy	Left hippocampus	Y	fc = 1 MHz; P = 0.35 MPa; PL = 10 ms; PRF = 1.25 kHz	↑ Iba1+	IF	Morse et al., 2019
Healthy	Not specific	Y	fc = 0.25 MHz; P = 200, 400 kPa; PL = 10 ms; PRF = 2 Hz; T = 2 min	↑ Iba1+ CD68+	IF	Pouliopoulos et al., 2021
Healthy	Cerebral cortex	Y	fc = 589.636 kHz; P = 0.3 MPa; PL = 10 ms	↑ Iba1+	IF	Kovacs et al., 2017
BV-2 cells	–	N	fc = 1 MHz; I = 30 mW/cm^2^; PL = 5 ms; PRF = 100 Hz; T = 15 min	↓ TNF-α, IL-1β, IL-6	WB	Chang et al., 2020
BV-2 cells	–	N	fc = 1 MHz; I = 30 mW/cm^2^; PL = 2 ms; PRF = 100 Hz; T = 15 min	↑ IL-10, Ym1	RTqPCR	Hsu et al., 2023

^*a*^FUS and FUS + microbubble parameters include fc, center frequency of the transducer in kHz or MHz; P, peak negative pressure of the transducer in kPa or MPa; I, intensity of the transducer in mW/cm^2^; PL, pulse length measured in microseconds (us) or milliseconds (ms); PRF, pulse repetition frequency measured in Hz; and T, time.

^*b*^Changes in microglial activity include Iba1, ionized calcium binding adaptor molecule 1; Aβ, amyloid beta; IL-1β, interleukin 1 beta; TMEM119, transmembrane protein 119; IL-10, interleukin 10; TNF-α, tumor necrosis factor alpha; BDNF, brain-derived neurotrophic factor; NK-κB, nuclear factor kappa B; CD68, cluster of differentiation 68; IL-6, interleukin 6; Ym1, chitinase-like protein 3 and increase (↑) and decrease (↓) in expression.

^*c*^Measurements include IF, immunofluorescence; ELISA, enzyme-linked immunosorbent assay; IHC, immunohistochemistry; RTqPCR, reverse transcription-quantitative polymerase chain reaction; and WB, western blots.

Morphological changes could enable microglia to approach vessels and respond to BBB disruption after FUS + microbubbles. Increased BBB permeability caused by systemic inflammation after the injection of LPS led to microglial morphology changes that resemble capillary-associated microglia (Bisht et al., 2021), including decreased numbers of microglial processes and increased soma sizes in mice (Haruwaka et al., 2019; Figure 2C). Similarly, hippocampal microglia in male mice exhibited minor reductions in the length and number of branches at 7 days after FUS + microbubbles (Silburt and Aubert, 2022). Remarkably, these differences in microglial processes became more pronounced when cells were spatially organized into morphological clusters (Silburt and Aubert, 2022). Thus, the effects of FUS + microbubbles on hippocampal microglia are heterogenous and spatially distributed at later timepoints. This could indicate that subsets of cells are more responsive to FUS + microbubbles. When the same methodology was applied to an aged mouse model of AD pathology (TgCRND8), focal microglia were found to overlap with Aβ plaques, suggesting a crosstalk between AD pathology and FUS + microbubbles sensitivity (Silburt and Aubert, 2022). To establish the directionality of this relationship, it would be important to characterize the responses of microglia in steady-state conditions, in particular, at early timepoints following FUS + microbubbles, when changes in microglial function could aid or hinder the return of BBB homeostasis. Moreover, as the integrity of the BBB is affected by sex, brain region and age, it follows that the context inhabited by microglia can affect the outcomes of BBB modulation after FUS + microbubbles.

### 4.2 Changes in cell number

The density and distribution of microglia varies across regions, subregions and even layers in the CNS, and is tightly balanced to support all the homeostatic functions of these cells (Khakpour et al., 2022). Microglia rearrange 10–15% of their cortical distribution daily in adult mice (Hammond et al., 2021) and this reorganization is further increased in response to stressors, e.g., seizures and laser injury (Eyo et al., 2018). In AD pathology, microglia proliferate and migrate toward Aβ plaques, shielding them to prevent further growth (Hammond et al., 2021). In homeostatic and pathological conditions, microglial density and distribution changes are stimulated by microenvironmental cues, which cause proliferation, migration and cell death (Khakpour et al., 2022). Disruption of the BBB permeability can influence the distribution of microglia by attracting cells toward vessels, potentially impacting their parenchymal surveillance. In male mice modeling systemic lupus erythematosus, cortical BBB disruption led to a decrease in the density of parenchymal Iba1+ cells, which was associated with increased expression of tight junction protein CLDN5 (Haruwaka et al., 2019). Similarly, FUS + microbubble BBB modulation can impact the distribution of microglia, although current analyses lack standardization, and it is particularly challenging to compare results generated by different research groups (Figure 2D). Two days after 0.25 MHz FUS + microbubble treatment in the pre-frontal cerebral cortex of adult non-human primates, at 0.8 MPa, but not 0.4 MPa, Iba1+ CD68+ cells migrated toward blood vessels. However, at both acoustic pressures, no changes in density were found 18 days after the treatment (Pouliopoulos et al., 2021). Changes in microglial density were not observed in the cerebral cortex of female and male adult TgCRND8 mice, after 0.5 MHz FUS + microbubbles at 0.3 MPa regardless of the proximity to Aβ plaques (Jordão et al., 2013). However, a variable reduction in the nearest neighbor density was observed 7 days after FUS + microbubbles targeting the hippocampus of male mice (Silburt and Aubert, 2022). The nearest neighbor distance represents the average distance between individual cells and their closest neighbor, thus, indicates the overall proximity between cells (González Ibanez et al., 2019).

In addition, 7 weeks after one or 6 weekly FUS + microbubble treatments (0.5 MHz at 0.3 MPa and 0.5 MPa), the Iba1+ area increased in the cerebral cortex of female rats, as did the number CD6+ cells (Kovacs et al., 2018). Elevated numbers of chemokine (C-X3-C motif) receptor 1+ and chemokine (C-C motif) receptor 2 cells, likely microglia, was similarly observed in a mouse model of glioma (GL26) receiving two or three 1.5 MHz FUS + microbubbles treatments at 0.5 MPa (Zhang et al., 2023). In an animal model of autoimmune encephalomyelitis immunized with recombinant myelin oligodendrocyte glycoprotein, FUS + microbubbles treatment was also associated with increased Iba1+ and transmembrane protein 119 (TMEM119)+ clusters of microglia at 12 days in young adult female mice (Schregel et al., 2021). These findings suggest that changes in the density and distribution of microglia in rodents can persist several days after one or multiple FUS + microbubble treatments. However, findings from similar investigations at earlier timepoints and in steady-state conditions are not yet available. Moreover, it is unclear if biological sex, brain region or age impact the outcome of FUS + microbubble treatments on microglial density and distribution. Notably, 4 h after FUS alone, fewer Iba1+ cells were observed in the substantia nigra pars compacta of female rats with toxin-induced dopaminergic cell depletion (Song et al., 2022). Both shorter duration (0.3 s) and longer duration (15 s) FUS pulses did not significantly affect the Iba1+ cell density in the cerebellum of adult female mice (Baek et al., 2021). It is possible that pre-existing inflammation may lead to higher likelihood of having microglia modify their density or distribution following FUS alone, warranting further research.

### 4.3 Changes in phagocytic activity

Microglia, the primary phagocytes in the brain, are involved in the clearance of apoptotic and infected cells, synapses, and misfolded proteins, such as Aβ, during both health and disease conditions (Galloway et al., 2019). Previous research has suggested that FUS alone can facilitate the removal of Aβ plaques by modulating microglial phagocytosis (Jordão et al., 2013; Bobola et al., 2020; Figure 2E). For instance, Bobola et al. (2020) reported that 2 MHz FUS pulsed at 40 Hz acutely cleared nearly 50% of plaques by promoting the co-localization of microglia with plaques in the hippocampus of male 5xFAD mice. With microbubbles, treatment of 0.5 MHz FUS at 0.3 MPa in TgCRND8 four-month-old mice significantly reduced plaque pathology with increased expression of Iba1+ cells in the treated hippocampus compared to the control (Jordão et al., 2013). Of note, in AD, microglia were located in close proximity to Aβ, enhancing clearance that might contribute to the observed reduction in plaque pathology (Marlatt et al., 2014). Moreover, 1.5 MHz FUS + microbubbles at 0.45 MPa in rTG4510 mice, a model of tauopathy, resulted in a reduction of tau pathology in the hippocampus (Karakatsani et al., 2019). In this study, an enhanced BBB permeability might have induced an immune response by promoting CD68 expression resulting in tau clearance in the hippocampus (Karakatsani et al., 2019). Furthermore, an antibody against Aβ protein delivered with 835 kHz FUS + microbubbles at 0.33 MPa resulted in higher Iba1 protein expression, which was associated with a reduction in plaques and less synaptic loss in the hippocampus of APP male mice, compared to FUS + microbubbles or intravenous antibody injection alone (Sun et al., 2021). In general, these studies hold clinical importance as FUS stimulation alone or with microbubbles can modulate microglial phagocytosis (Figure 2E). However, further *in vivo* research is needed to directly elucidate the precise mechanisms by which FUS or FUS + microbubbles influence microglial phagocytosis.

### 4.4 Changes in cytokine secretion

Excessive cytokine production induced by microglia is recognized as a pivotal mechanism in the pathogenesis of neurodegenerative diseases, and, therefore, the modulation of this immune response is regarded as a promising therapeutic strategy (Muzio et al., 2021; Figure 2F). Several studies have reported that FUS can modulate microglial cytokine expression and enhance anti-inflammatory properties even in the absence of microbubbles. 1 MHz FUS at 528 mW/cm^2^ can protect the degeneration of dopaminergic neurons and suppress microglia-induced IL-1β protein expression associated with the progression of Parkinson’s disease pathology in female rats using 6-OHDA (Song et al., 2022). Mice with middle cerebral artery occlusion, a model of ischemic stroke, treated with 0.5 MHz FUS at 120 mW/cm^2^ for 7 days showed increased IL-10 gene expression, as well as the number of Iba1+ Arg1+ expressing microglia (Wang et al., 2021). Additionally, after FUS, a reduction in stroke-induced brain atrophy was observed, which correlated with improved outcomes on the elevated body swing test and corner test, associated with motor function (Wang et al., 2021). These results suggest that FUS can modulate microglial activity and enhance recovery after brain injury. In male LPS-treated mice stimulated with 1 MHz FUS at 528 mW/cm^2^, the protein expression of TNF-α and IL-1β was reduced, while brain-derived neurotrophic factor (BDNF) levels were enhanced in the hippocampus (Chen et al., 2019). Moreover, *in vitro* studies have provided additional molecular and cellular understanding on how microglia respond to FUS in a controlled environment which may not be achieved in *in vivo* models. Following stimulation with ultrasound at 1 MHz and 30 mW/cm^2^, the protein expression of TNF-α and IL-1β was reduced in LPS-treated BV-2 cells, an immortalized mouse microglia-like cell line (Chang et al., 2020). Consistent with this finding, Hsu et al. (2023) reported that ultrasound can prevent excessive inflammation by enhancing the gene expression of IL-10 and chitinase like-3 (Ym1) associated with tissue repair in BV-2 cells treated with LPS. However, investigations using more complex models are needed to confirm these *in vitro* findings. Emerging *in vitro* models, such as induced pluripotent stem cell-derived human microglia, can allow disease and human-specific responses to be studied, which may not be accurately represented in primary or immortalized cultures.

## 5 Outstanding questions

### 5.1 Nanometric changes following FUS + microbubbles

Ultrastructure analysis by electron microscopy can provide rich snapshots of microglial responses to their microenvironment, notably in contexts of inflammation (Savage et al., 2018, 2019). However, to our knowledge, no study has yet accessed the nanometric outcomes of FUS or FUS + microbubbles on these cells. Microglia communicate closely with blood vessels and synaptic elements in the CNS (Šimončičová et al., 2022) making it possible that after BBB modulation with FUS + microbubbles, the extravasation of blood molecules and endothelial chemokine release disturb these crosstalks (Savage et al., 2019; Pouliopoulos et al., 2021; Figure 2). Following LPS intraperitoneal or subcutaneous injection, typically associated with BBB disruption, there were more associations between parenchymal Iba1+ cells and vessel surfaces in the hippocampus, cerebral cortex and thalamus (Bowyer et al., 2020; Erickson et al., 2023). Once microglia are in contact with the BBB, the functional outcomes are context dependent. In a mouse model of lupus, markedly presenting increased BBB permeability, microglia processes were suggested to project through the basement membrane at day one and to contain phagocytic inclusions with astrocytic content at day seven, which could indicate an effort to seal the leaky BBB but also engulf astrocytic endfeet (Haruwaka et al., 2019). This suggested a temporal shift from adaptive to maladaptive microglial responses due to a sustained BBB permeability (Haruwaka et al., 2019). Considering the transient nature of BBB modulation after FUS + microbubbles, it is plausible that microglia-BBB interactions are not significantly affected (Figure 2C), although the interactions between microglia and synaptic elements may still be perturbed (Figure 2G). In the hippocampus CA1 of adult female and male mice, 24 h after treatment with LPS, Iba1+ processes were more frequently in contact with synaptic clefts, presynaptic terminals and dendritic spines during sickness behavior (Savage et al., 2019). Thus, alterations in microglial-neuronal contacts after FUS + microbubbles could contribute to modifying behavior. In addition, inflammatory insults, such as the one elicited after BBB modulation, are typically accompanied by ultrastructural changes in microglial mitochondria to comply with their higher metabolic rates and reactive oxygen species production (Katoh et al., 2017; El Hajj et al., 2019; Savage et al., 2019; St-Pierre et al., 2022). Notably, microglia showing ultrastructural signs of stress, such as mitochondria dystrophy, interacted less with the vasculature in the hippocampus of aged male APP-PS1 mice (St-Pierre et al., 2022). Acute inflammation induced by FUS + microbubbles might reversibly cause a similar cellular stress in microglia at the ultrastructural level (Figure 2H), an important research avenue for future investigation.

### 5.2 Baseline responses to FUS and FUS + microbubbles

Previous investigations in healthy brain regions have provided evidence that microglia are responsive to the mechanical energy and cavitation associated with FUS + microbubbles (Kovacs et al., 2017; McMahon and Hynynen, 2017; Sinharay et al., 2019). Following 1.1 MHz FUS + microbubble at 0.42 MPa, Choi et al reported an increased number of Iba1+ cells in the treated thalamus of male mice compared to stimulation at 0.25 MPa. Mice exposed to a higher acoustic pressure of 0.42 MPa, were associated with amoeboid Iba1+ cells and an upregulation of NK-κB pathway genes (Choi et al., 2022). In addition, exposure from blood-derived proteins such as albumin may have a putative role in modulating microglial function and upregulating pro-inflammatory genes (Ranaivo and Wainwright, 2010). One way to limit the extravasation of albumin after FUS + microbubbles would be to reduce the extent of the BBB permeability. Rapid short-pulses of FUS + microbubbles at an acoustic pressure of 0.35 MPa have been shown to increase the permeability of the BBB for < 10 min and led to a 3.4-fold less albumin being released into the brain when compared with longer pulses in the hippocampus of female mice (Morse et al., 2019). Moreover, Sierra et al. (2017) observed a higher density of microglial cells following 1.5 MHz FUS + microbubbles at an acoustic pressure of 0.75 MPa in the sonicated region compared to the control. Notably, in some contexts, e.g., Aβ accumulation, robust initiation of an immune response could be desirable to promote phagocytosis (Galloway et al., 2019). However, an excessive increase in microglial activity, particularly when associated with an increased production of pro-inflammatory cytokines could result in neuronal death (Glass et al., 2010; Leinenga et al., 2019). Understanding the baseline changes in microglial number, form and function after FUS + microbubbles and FUS could aid in adapting this technology to optimally modulate the surveillant and phagocytic activity of microglia toward disease resolution.

### 5.3 FUS and FUS + microbubbles in regions impacted by neurodegeneration

Many studies strongly support that microglia are involved in AD pathology and can efficiently elicit Aβ clearance through phagocytosis (D’Andrea et al., 2004; Pan et al., 2011). However, a deficiency in this function, as seen in the later stages of AD, can accelerate the progression of the disease (Ewers, 2020). AD presents an enormous therapeutic challenge as the currently available treatments only offer temporary symptomatic relief without any effect on disease progression. Over the last decades, multiple well-conducted clinical trials have failed due to excessive side effects, drug toxicity and a lack of changes in behavioral and cognitive functions (Asher and Priefer, 2022). Several studies have reported the therapeutic potential of FUS + microbubbles to alleviate the pathogenesis of AD in various *in vivo* models (Jordão et al., 2013; Burgess et al., 2014; Poon et al., 2018; Karakatsani et al., 2019; Leinenga et al., 2019). A significant improvement in memory tasks has been observed following FUS + microbubbles at 0.996 MHz and 0.64 MPa in the hippocampus of 3xTg-AD mice that display both amyloid and tau pathology (Shen et al., 2020). Of note, this effect was associated with increased microglial phagocytosis of Aβ deposits and improved axonal health in CA1 and CA3 neurons (Shen et al., 2020). Scanning ultrasound (SUS), i.e., transcranial focused ultrasound targeting multiple spots in the brain, with microbubbles at 1 MHz and 0.7 MPa, was also shown to reduce plaques through increased Aβ in the lysosomes of microglia from APP23 mice (Leinenga and Götz, 2015). However, SUS without microbubbles (1 MHz at 0.7 MPa) was not sufficient to remove Aβ load in APP23 mice suggesting that the increase in BBB permeability and the extravasation of immunoglobulin or albumin could modulate microglia to phagocytose the plaques (Leinenga et al., 2019). FUS + microbubbles treatment can remarkably promote clearance of Aβ by microglia and alleviate neuronal plaque accumulation, further supporting the clinical value of this technology (Shen et al., 2020).

### 5.4 Single and repeated FUS + microbubbles treatments

However remarkable in its effects, it is still unclear whether one or repeated FUS treatments, with or without microbubbles, would be enough to delay or prevent disease progression in the CNS. The main concern would be whether frequent exposure to acoustic radiation and cavitation could have undesirable effects in the CNS. Notably, FUS + microbubbles delivered once a week for 3 weeks to the hippocampus of APP male mice, resulted in improved cognitive behavior, without changes in fear learning, memory or locomotor activity. These effects were combined with a reduction in synapse loss in the hippocampus of APP male mice at 18 days after the last treatment (Sun et al., 2021). Despite identifying higher Iba1 protein expression, it remains unclear if the homeostatic functions of microglia were maintained or restored as a consequence of the BBB modulations. Studies so far have indicated elevations in microglial markers after one or multiple FUS + microbubble treatments in murine models (Kovacs et al., 2018; Wang et al., 2021; Zhang et al., 2023). Similarly, increased [18F]DPA-714 binding to the translocator protein, present in microglia and typically upregulated with inflammation, was observed after a single, two and six weeks of pulsed FUS + microbubbles exposures in the cerebral cortex and hippocampus of female rats (Sinharay et al., 2019). However, FUS without microbubbles has led to context-dependent findings, at least in terms of Iba1 expression. While restored upon five daily treatments with low-intensity pulsed ultrasound alone in a Parkinson’s disease pathology model (Song et al., 2022), the number of Iba1+ cells increased in male mice treated with FUS alone for seven consecutive days after middle cerebral artery occlusion (Wang et al., 2021). It stands that further investigation is required to understand the aftermath of Iba1 modulation, such as assessing its functional impact on synaptic plasticity (Lituma et al., 2021). Despite lack of clinical investigations on microglia, up to three consecutive FUS + microbubbles treatments in clinical trials have only suggested mild to moderate adverse effects including headaches, vagal responses and musculoskeletal pain (Lipsman et al., 2018; Abrahao et al., 2019; Mainprize et al., 2019; Meng et al., 2021; Park et al., 2021). Future positron emission tomography studies could aid in understanding the outcome of multiple FUS + microbubbles on microglia (Šimončičová et al., 2022).

### 5.5 Microglial diversity

Most studies investigating microglia after FUS or FUS + microbubbles utilize Iba1 expression, combined or not with morphological features. However, there are important limitations with the current findings available (Gonçalves de Andrade et al., 2021; Paolicelli et al., 2022; Šimončičová et al., 2022). Firstly, in addition to microglia, Iba1 is expressed by border-associated macrophages, such as macrophages from the choroid plexus, meninges and perivascular space, as well as peripheral monocytes (Gonçalves de Andrade et al., 2021). As previously discussed, immune cell infiltration after FUS + microbubbles is rare but possible, depending on the applied parameters. Thus, future studies in the FUS field using specific markers to differentiate immune populations in the treated brain would be useful. Secondly, fluctuations in Iba1 expression are not enough to inform microglial function. Iba1 expression is related to cell membrane rearrangements, with a plausible impact on phagocytosis, ATP-induced ramification and motility (Lituma et al., 2021). However, this marker cannot reliably predict beneficial or detrimental microglial functional outcomes (Gonçalves de Andrade et al., 2021; Paolicelli et al., 2022; Šimončičová et al., 2022). Advances in our understanding of microglia indicate that the consequences of their changes, notably in gene and protein expression levels, are determined by their microenvironment. According to their specific context, including species, sex, brain region, and organisms’ age, microglia vary in cellular states in the brain parenchyma. These states have distinct layers of complexity (e.g., epigenetic, transcriptional, translational, and metabolic signatures), which define their phenome (i.e., motility, morphology, and ultrastructure) and function (Paolicelli et al., 2022). Inflammatory and anti-inflammatory balances are tightly regulated by microglial populations, such that increases in pro- or anti-inflammatory molecules can be protective to the CNS. As a result, investigations after FUS or FUS + microbubbles would benefit from including omics, phenotypic, and functional microglial assays to characterize the impact on these cells.

## 6 Discussion

Central nervous system diseases include a spectrum of debilitating conditions that affect the brain and spinal cord, such as tumors, ischemic stroke, epilepsy, and neurodegenerative diseases like AD (Feigin et al., 2020). The incidence of AD and other dementias increases with age, and approximately 50 million individuals worldwide are affected by this condition (Nichols et al., 2022). This number is set to rise substantially to 152 million by 2050 – causing a significant individual and societal burden (Nichols et al., 2022). However, the development of effective therapies to treat or prevent dementia and other neurodegenerative diseases remains a challenge that is amplified by the BBB. FUS + microbubbles can transiently overcome the BBB and modulate microglial structure and function, with beneficial outcomes on the onset and progression of neurodegeneration (Blackmore et al., 2023). Similarly, acoustic radiation from FUS without microbubbles can activate mechanosensitive ion channels in microglia and modify their responses.

This review outlines evidence supporting that FUS with and without microbubbles may trigger acute inflammatory responses that alter the shape, organization, phagocytic activity, cytokine and chemokine secretion of microglia. Current findings in the field have built a solid foundation, but many questions remain unanswered, particularly with respect to the microglia-BBB and microglia-neuron interactions. Furthermore, the inflammatory and phagocytic pathways triggered following FUS and FUS + microbubbles are still elusive. We highlight important factors to consider in future investigations on the properties and functions of microglia after FUS, including the inflammatory status of the targeted region, baseline microglial responses and their irrevocable diversity. Studies so far are encouraging as they suggest, for instance, that microglia co-localize with misfolded proteins, enhancing their clearance. However, given the broad role microglia play in CNS health, it will be important to ensure that FUS and FUS + microbubbles can diminish pathological functions of microglia and promote their homeostatic roles.

## Author contributions

SG: Conceptualization, Writing – original draft, Writing – review and editing. EG: Conceptualization, Writing – original draft, Writing – review and editing. RK: Writing – original draft, Writing – review and editing. PMM: Supervision, Writing – review and editing. IA: Funding acquisition, Resources, Supervision, Writing – review and editing. M-ÈT: Conceptualization, Funding acquisition, Resources, Supervision, Writing – review and editing. SVM: Conceptualization, Funding acquisition, Resources, Supervision, Writing – review and editing.
